# Complete mitochondrial genome of the feather star *Cenometra bella* (Hartlaub, 1890) (Crinoidea: Colobometridae)

**DOI:** 10.1080/23802359.2022.2080026

**Published:** 2022-06-07

**Authors:** Jia Jin Marc Chang, Yin Cheong Aden Ip, Danwei Huang

**Affiliations:** a Department of Biological Sciences, National University of Singapore, Singapore; b Centre for Nature-based Climate Solutions, National University of Singapore, Singapore; c Lee Kong Chian Natural History Museum, National University of Singapore, Singapore; d Tropical Marine Science Institute, National University of Singapore, Singapore

**Keywords:** Crinoids, Echinodermata, genome skimming, mitogenome, phylogeny

## Abstract

The complete mitochondrial genome of the feather star *Cenometra bella* was sequenced in this study. The mitogenome is 15,872 bp in length, with 13 PCGs, 22 tRNA, and two rRNA, and nucleotide composition was as follows: 24.38% A, 47.79% T, 11.16% C, and 16.68% G. Phylogenetic analyses place *C. bella* as closely related to *Stephanometra indica*, consistent with previous inferences.

As suspension feeders that regulate primary production on reefs (Baumiller 2008), crinoids constitute important components of marine communities. They comprise more than 600 nominal extant species, yet remain the least studied group among extant echinoderms (Rouse et al. 2013). This is evident in the paucity of genetic resources (i.e., mitogenome sequences) available for Crinoidea, especially Colobometridae, on public sequence databases such as GenBank (last accessed 12 October 2021). Here, we sequenced the mitogenome of *Cenometra bella* (Hartlaub, 1890) (Crinoidea: Colobometridae), a feather star species that is widely distributed across the tropical waters of the Indo-West Pacific Ocean (Zmarzly 1984; Arguelles et al. 2010; Britayev and Mekhova 2011; Sadhukhan and Raghunathan 2012; Tay 2015).

The *C. bella* specimen was collected on 15 July 2020 along the dive trail at Sisters’ Islands Marine Park, Singapore (1°12′46.77″N, 103°50′11.07″E). The specimen was previously DNA barcoded for COI (Chang et al. 2020). The voucher (ZRC.ECH.1687) is housed at the Zoological Reference Collection, Lee Kong Chian Natural History Museum, Singapore (Curator: Ms Iffah Iesa, nhmii@nus.edu.sg). We re-extracted genomic DNA from the cirri using DNeasy Blood and Tissue Kit (Qiagen, Hilden, Germany) following manufacturer’s protocols. Genomic DNA was sheared to ∼300 bp, and a library was prepared with NEBNext^®^ Ultra^TM^ II DNA Library Prep Kit (New England Biolabs, Ipswich, MA) (see also Quek et al. 2019). Sequencing was outsourced to the Genome Institute of Singapore and performed on ∼25% of an Illumina MiSeq^TM^ run (250 bp, paired-end).

Our genome skimming bioinformatic pipeline followed Chang et al. (2022). A total of 7,888,554 raw reads were first trimmed using fastp v0.20.1 (Chen et al. 2018), before piping into GetOrganelle v1.7.5 (Jin et al. 2020) for mitogenome assembly. Based on past phylogenetic results from Rouse et al. (2013), as well as availability of mitogenome sequences on GenBank (last accessed 12 October 2021), *Stephanometra indica* (MF966246) was found to be most closely related to *C. bella* and, hence, selected as the seed sequence for mitogenome assembly. We successfully retrieved the complete *C. bella* mitogenome at 62× sequencing coverage. The mitogenome was then annotated using MITOS2 web server (Bernt et al. 2013; Donath et al. 2019) (RefSeq 81 Metazoa, Genetic Code 9) for protein-coding genes (PCGs), transfer and ribosomal RNA genes. Finally, the PCG annotations were manually curated according to Quek et al. (2021) to ensure that annotations were in frame and accurate. The eventual *C. bella* mitogenome sequence was 15,872 bp in length, and the overall nucleotide composition was as follows: 24.38% A, 47.79% T, 11.16% C, and 16.68% G. All 13 PCGs, 22 tRNA and two rRNA genes were recovered. The start codon ATG was featured in all 13 PCGs, while TAA was the most common termination codon (*n*=10), followed by TAG (*n*=3). We observed that the gene order was identical to that of *S. indica* (MF966246).

For phylogenetic reconstruction, we downloaded eight other publicly available Crinoidea mitogenomes from GenBank. We also included one Asteroidea and two Ophiuroidea mitogenomes as outgroups. The PCGs and ribosomal genes were extracted and aligned separately by gene using MAFFT v7.407 (Katoh and Standley 2013) before concatenation to form a sequence matrix 15,058 bp in length. The matrix was partitioned by gene, and best-fitting evolutionary models for each partition were evaluated using ModelTest-NG v0.1.7 (Darriba et al. 2020). The gene model information was then used for maximum-likelihood and Bayesian analyses. Maximum-likelihood was performed using RAxML-NG v1.0.3 (Kozlov et al. 2019), with 200 starting trees (100 random and 100 parsimony-based), and node supports were quantified with 1000 bootstrap pseudoreplicates. The Bayesian inference was performed using MrBayes v3.2.7 (Ronquist et al. 2012), in which we initiated four Markov chains Monte Carlo (MCMC) of 10,000,000 generations implemented over two runs, and sampled one tree per 100 generations. MCMC convergence was assessed in Tracer v1.7 (Rambaut et al. 2018), after discarding the first 10,001 trees as burn-in. The resulting trees were congruent and achieved maximal support at most nodes. *Cenometra bella* was recovered as sister to *S. indica* (Figure 1), a pattern consistent with previous phylogenetic work that showed *Cenometra* and *Stephanometra* as the closest relatives among genera examined here (Rouse et al. 2013; Taylor 2015).

**Figure 1.**
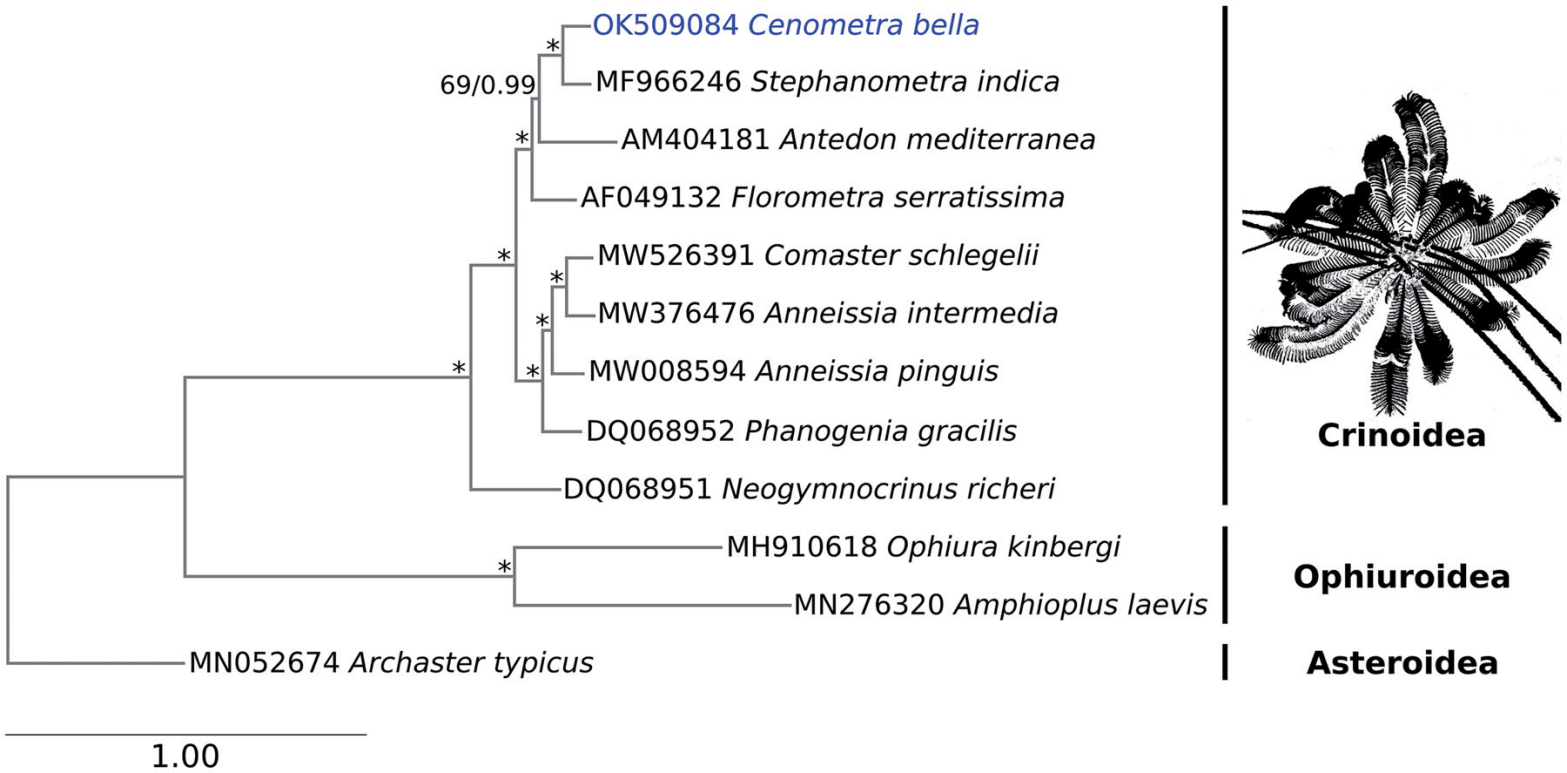
Phylogenetic reconstruction of Crinoidea, using a concatenated matrix of 13 protein-coding genes and two ribosomal RNA genes. A sketch of the *Cenometra bella* sample is also shown. Asteroidea and Ophiuroidea mitogenomes were used as outgroups. Bootstrap support and posterior probability values are shown adjacent to each node, before and after the slash, respectively; nodes with 100 bootstrap support and 1.00 posterior probability are labeled with an asterisk (*).

## Data Availability

The complete *Cenometra bella* mitogenome has been uploaded to NCBI GenBank under accession number OK509084. The associated raw sequence reads have been deposited at NCBI under BioProject PRJNA770161, BioSample SAMN22210553, and SRA SRR16292889.
